# Improving IL12 immunotherapy in glioblastoma by targeting the long noncoding RNA *INCR1*

**DOI:** 10.1007/s11060-025-04978-2

**Published:** 2025-03-04

**Authors:** Shikha Saini, Josephina A. M. A. Gadet, Gordon J. Freeman, E. Antonio Chiocca, Marco Mineo

**Affiliations:** 1 Harvey W. Cushing Neuro-oncology Laboratories (HCNL), Department of Neurosurgery, Harvard Medical School and Brigham and Women’s Hospital, Boston, MA 02115 USA; 2 Faculty of Medicine, Amsterdam University Medical Centers, Location AMC, Amsterdam, The Netherlands; 3 Department of Medical Oncology, Dana-Farber Cancer Institute, Harvard Medical School, Boston, MA 02115 USA

**Keywords:** GBM, Immunotherapy, IL12, *INCR1*, PD-L1

## Abstract

**Purpose:**

The potent antitumor effects of interleukin 12 (IL12) gene therapy in glioblastoma (GBM) are significantly attenuated by the highly immunosuppressive microenvironment and the upregulation of the PD-1/PD-L1 immune checkpoint. However, combining IL12 gene therapy with PD-1/PD-L1 inhibitors failed to improve efficacy. This study aims to assess the effects of silencing the immunosuppressive long noncoding RNA *INCR1* when combined with IL12 therapy.

**Methods:**

RNAscope in situ hybridization was performed to analyze *INCR1* and *PD-L1* expression in tumor tissues from GBM patients pre- and post-IL12 gene therapy. Quantitative PCR was used to analyze immunosuppressive gene expression in patient-derived GBM cells co-cultured with immune cells stimulated with IL12. The effects of *INCR1* and *PD-L1* silencing on the expression of immunosuppressive genes were evaluated by RNA sequencing. 3D-cytotoxicity assays were performed to assess the activity of immune cells against GBM tumor cells.

**Results:**

*INCR1* and *PD-L1* expression was upregulated in tumor tissue from GBM patients treated with IL12 gene therapy compared to the tumor tissue of the same patients before the IL12 treatment. Co-culture of patient-derived GBM cells with IL12-stimulated immune cells increased the expression of several immunosuppressive genes. Knocking down *INCR1* was more effective than silencing *PD-L1* in reducing the expression of multiple immunosuppressive genes. *INCR1* silencing improved IL12-mediated immune cell antitumor activity compared to monoclonal antibodies targeting the PD-1/PD-L1 immune checkpoint signaling.

**Conclusion:**

*INCR1* silencing affects more immune evasive pathways than PD-L1. Targeting *INCR1* may represent a valid approach to improve the efficacy of IL12 therapy in GBM.

**Supplementary Information:**

The online version contains supplementary material available at 10.1007/s11060-025-04978-2.

## Introduction

Glioblastoma (GBM) is the most common and aggressive primary cancer of the brain. Despite advances in therapeutic intervention, including maximal tumor resection, chemotherapy, and radiotherapy, GBM patient median survival remains approximately 20.9 months [1]. Immunotherapy represents a promising approach to overcome current therapeutic challenges and improve outcomes for GBM patients [2]. Several immunotherapies have demonstrated efficacy against hematologic and solid cancers [3, 4], and there is strong interest in applying these strategies to GBM. However, clinical trials of immunotherapies against GBM have been conducted with minimal success due to the highly immunosuppressive microenvironment that makes this tumor resistant to immunotherapy [5]. Mechanisms related to this state of immunosuppression are multifactorial but at the cellular level, this immunosuppressive milieu features an abundance of myeloid cells and a paucity of tumor-infiltrating cytotoxic CD8 + and helper CD4 + T cells [6, 7]. To revert this cellular imbalance in the GBM microenvironment, we and others have been utilizing direct intra- or peri-tumoral administration of gene therapies that express potent immune activators, both preclinically and clinically [8–10]. Interleukin 12 (IL12) is one of the most potent cytokines mediating antitumor immune responses [11]. IL12 enhances natural and adaptive immunity, potently stimulates the production of interferon-gamma (IFNγ) through activating NK cells and cytotoxic T cells, and alters the tumor microenvironment [12]. We have recently found that peritumoral administration of a regulatable IL12 gene therapy in subjects with recurrent GBM showed increased inflammatory GBM infiltrates, including increased numbers of CD8 + T cells and elevated levels of tumor IFNγ [13]. However, this also was coupled with an increase in tumor programmed cell death ligand 1 (PD-L1) and programmed cell death 1 (PD-1)-positive cells, characteristic of chronic exhaustion and immune escape. Notably, IL12 gene therapy coupled with neo-adjuvant immune checkpoint inhibition of PD-1 did not show improved efficacy [14], suggesting that inhibition of a single immune checkpoint is not sufficient to overcome evasion from IL12-induced immunity. Identifying a common pathway that regulates the expression of multiple immunosuppressive genes will, therefore, allow the development of alternative strategies to overcome the current limitations of IL12 gene therapy.

Long noncoding RNAs (lncRNAs) are transcripts longer than 200 nt that lack protein-coding potential [15, 16]. They act as major regulators of a wide range of cellular processes in both physiological conditions and diseases [16, 17]. LncRNAs, through interaction with DNA, proteins, or other RNAs, can regulate the expression of multiple genes involved in specific biological functions [18]. We have recently identified the interferon-stimulated noncoding RNA 1 (*INCR1*) as a novel lncRNA transcribed from the *PD-L1* locus and showed that *INCR1* is highly inducible in tumor cells stimulated with IFNγ [19]. We demonstrated that *INCR1*, through the interaction with the ribonucleoprotein HNRNPH1, regulates the expression of the neighboring genes *PD-L1* and *JAK2*. The regulation of *JAK2*, in turn, leads to an indirect effect on *STAT1* and the expression of different immunosuppressive molecules [19]. While targeting *INCR1* increased immune-mediated tumor cell death, the effects of *INCR1* silencing on the efficacy of IL12 immunotherapy remain unknown. In this study, we analyzed the expression of *INCR1* in GBM patients treated with regulatable IL12 gene therapy. We investigated the effects of *INCR1* silencing on IL12-mediated antitumor functions of immune cells. We also compared *INCR1* targeting to immune checkpoint inhibitors. We believe our findings provide novel insights for the future development of therapies that target *INCR1* to improve IL12 gene therapy.

## Materials and methods

### Human specimens

Tumor tissue samples were obtained with the consent of the participants as approved by the Institutional Review Board (IRB) at the Dana-Farber Cancer Institute. Patient samples were processed for RNA in situ hybridization analysis.

### RNAscope in situ hybridization

RNAscope in situ hybridization was performed using RNAscope 2.5 HD Reagent Kit-RED (cat# 322360, ACD Bio) according to the manufacturer’s protocol. Briefly, tissue sections were deparaffinized by baking the slides for 1 h at 60 °C, followed by incubation in xylene and ethanol. Target retrieval was performed by submerging the slides in boiling Target Retrieval solution, followed by protease treatment for 30 min at 40 °C. Probes were then hybridized for 2 h at 40 °C, followed by RNAscope amplification and red chromogenic detection. The slides were counterstained with 50% hematoxylin I for 2 min at room temperature and mounted. The following RNAscope probes were used: INCR1 (cat#1078641-C1, ACD Bio), CD274 (PD-L1, cat# 600861, ACD Bio), NEAT1 (cat# 411531) as positive control probe, and negative control probe (cat# 310043). Images were acquired under a bright field at 60X magnification using NIS-elements software on a Nikon Ti microscope. Samples were analyzed by counting the percent of positive cells.

### Cell culture

Patient-derived primary GBM cells (G44, BT139, and G62 cell lines) were generated as previously described [20] and cultured as neurospheres in stem cell conditions using Neurobasal (Thermo Fisher Scientific) supplemented with Glutamine (Thermo Fisher Scientific), B27 (Thermo Fisher Scientific), 20 ng/ml epidermal growth factor (EGF) and fibroblast growth factor (FGF)-2 (PrepoTech). U251 cells were purchased from the American Type Culture Collection (ATCC) and cultured in DMEM (Thermo Fisher Scientific) supplemented with 10% fetal bovine serum (FBS, Sigma-Aldrich). Interleukin-12 (IL12, PeproTech) stimulation was performed at 50 ng/ml. Stable U251 *INCR1* knockdown was obtained as previously described [19]. *PD-L1* knockdown was performed by transfecting 50 pmol/well of Duplex siRNAs (hs.Ri.CD274.13.1, hs.Ri.CD274.13.2, Integrated DNA Technologies) for 6-well plates using Lipofectamine RNAiMAX (Thermo Fisher Scientific). *INCR1* knockdown using ASOs was performed by transfecting 100 pmol/well of GapmeR ASO (ASO#1 TCTCCCCTTGGTCTTT; ASO#2 CCCTCTAGTTGTAGTCTT; ASO#3 TCCCTAATAGTAAGCTGA) for 6-well plates using Lipofectamine 2000 (Thermo Fisher Scientific).

### 3D PBMC cytotoxicity assay

Peripheral blood mononuclear cells (PBMCs) were obtained from healthy human donors as approved by the IRB at the Brigham and Women’s Hospital. PBMCs were isolated using Ficoll Paque Plus (GE Healthcare Life Sciences) following the manufacturer’s instructions. 750 GFP positive control or *INCR1*-knockdown tumor cells were seeded in a round bottom low-attachment 96-well plate. Cells were allowed to form tumorspheres for 72 h. After tumorspheres were formed, twenty thousand PBMCs were added. Tumorspheres and PBMCs were co-cultured for 96 h with or without IL12 treatment, and isotype control antibody (Bethyl Laboratories), anti-PD-1 antibody (EH12) [21], or anti-PD-L1 antibody (29 F-2A3) [22]. Images were acquired using NIS-elements software on a Nikon Ti microscope, and changes in GFP intensity were measured using ImageJ.

### Quantitative real-time PCR analysis

Total RNA was extracted using TRIzol (Thermo Fisher Scientific), reverse transcribed using iScript cDNA Synthesis Kit (BioRad), and quantitative real-time PCR was performed using SYBR Green Master Mix (Applied Biosystem). 18 S expression level was used as control. The primers used throughout the study are listed in Table S1.

### RNA-seq and analysis of RNA-seq data

U251 were transfected with siRNA control or siRNAs targeting *PD-L1* and incubated for 48 h. After incubation, cells were stimulated with IFNγ for 24 h. RNA was extracted using TRIzol (Thermo Fisher Scientific). 1 µg of total RNA was used, and RNA libraries were prepared by poly(A) selection. Paired-end reads were sequences on a HiSeq System (Illumina) to achieve at least 40 million reads per sample. Libraries prepared from two independent experiments were analyzed. Raw data were trimmed and mapped using CLC genomics workbench version 22.0.2. Quality trimming was set at quality limit = 0.05, maximum number of ambiguities = 2. Trimmed reads were aligned to the human genome from the Ensembl release GRCh38. The mapping options were set as mismatch cost = 2, insertion cost = 3, deletion cost = 3, length fraction = 0.8, similarity fraction = 0.8, auto-detect paired distances, maximum number of hits for a read = 10. Expression setting was set as strand setting = both, library type setting = bulk, expression level = ignore broken pairs. CLC genomics workbench version 22.0.2 was used to normalize expression counts and to obtain differentially expressed genes between control and *PD-L1* knockdown cells.

### Pathway enrichment analysis

Pathway enrichment analysis was conducted using Gene Set Enrichment Analysis (GSEA) software version 4.1.0. Analyses in GSEA were performed on the counts per million generated in CLC genomics workbench. The analysis used the gene set database h.all.v2022.2.Hs.symbols.gmt (Hallmarks) with number of permutations set at 1000, collapse/remap to gene symbol = collapse, permutation type = gene_set, enrichment statistic = weighted, metric for ranking genes = Signal2Noise, gene list sorting mode = real, gene list ordering mode = descending, max size: exclude larger sets = 500, min size: exclude smaller sets = 15.

### Statistical analysis

Data are expressed as mean ± SD. Statistical analyses were performed using the unpaired two-tailed Student’s t-test from GraphPad Prism software. Differences were considered statistically significant at *P* < 0.05. For differential gene expression of the RNA sequencing data, significant genes were detected using CLC genomics workbench version 22.0.2-generated false discovery rate (FDR) values that were less than 0.01. In the GSEA analysis, enriched gene sets with an FDR less than 0.05 were considered significant.

## Results

### *INCR1* expression is upregulated in GBM patients treated with regulatable IL12 gene therapy

**Fig. 1:**
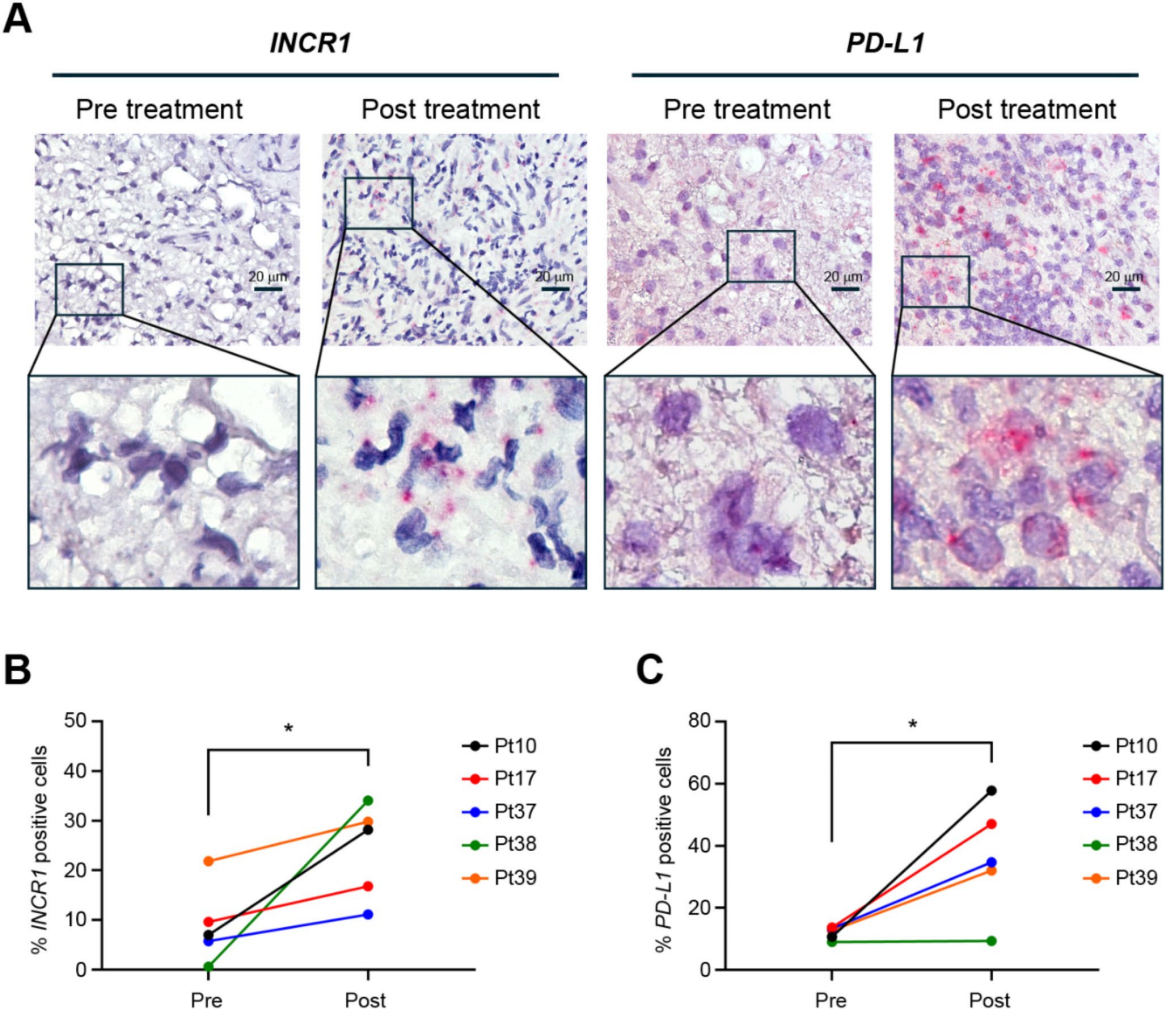
*INCR1* and *PD-L1* expression levels increase in GBM patients treated with regulatable IL12 gene therapy: (**A**)* INCR1* (left) and *PD-L1* (right) RNA scope staining of FFPE tissues from GBM patients pre- and post-IL12 gene therapy. Red dots are indicative of *INCR1* (left panels) and *PD-L1* (right panels) signals. Scale bar: 20 μm. **B-C** Quantification of *INCR1* (**B**) and *PD-L1* (**C**) positive cells in five different GBM patients pre- and post-IL12 therapy. Data were analyzed by paired t-test: *p < 0.05

To investigate the transcriptional profile of the lncRNA *INCR1* in GBM patients treated with IL12 immunotherapy, we performed RNAscope in situ hybridization on tumor tissues from five GBM patients (Pt10, Pt17, Pt37, Pt38, Pt39) before and after administration of IL12. Patients undergoing recurrent high-grade glioma resection received a fixed dose of replication-incompetent adenoviral Ad-RTS-hIL12 gene therapy followed by administration of the activator ligand, veledimex, which activates the transcription of the IL12 transgene. Details on patient characteristics and treatment modalities were previously published ( [13], Table S2). Hybridization of the tumor tissues with an *INCR1* probe showed an increased signal in all GBMs post-IL12 treatment compared to the signal before IL12 therapy (Fig. S1A, Fig. 1A). The increased signal was associated with a statistically significant increase in the number of *INCR1*-positive cells in the patients post-IL12 (Fig. 1B). No signal was observed when using a negative control probe, and all cells were stained by a probe targeting the lncRNA *NEAT1* that was used as a positive control (Fig. S1B). Since *INCR1* is transcribed from the *PD-L1* locus, the same tissues were next hybridized with a probe targeting the *PD-L1* mRNA. As shown in Fig. S1A and Fig. 1A, except for patient 38, the *PD-L1* signal increased in tumor tissues post-IL12 therapy. A significant increase in the number of *PD-L1*-positive cells in patients treated with IL12 (Fig. 1C) confirmed our previous immunofluorescence results using an antibody for PD-L1 (Fig. 3B, C in [13]). Together, these data indicate that regulatable IL12 gene therapy is associated with increased expression of *INCR1*, which may be involved in immunotherapy resistance.

### IL12 treatment induces the expression of multiple immunosuppressive genes in patient-derived GBM cells

**Fig. 2:**
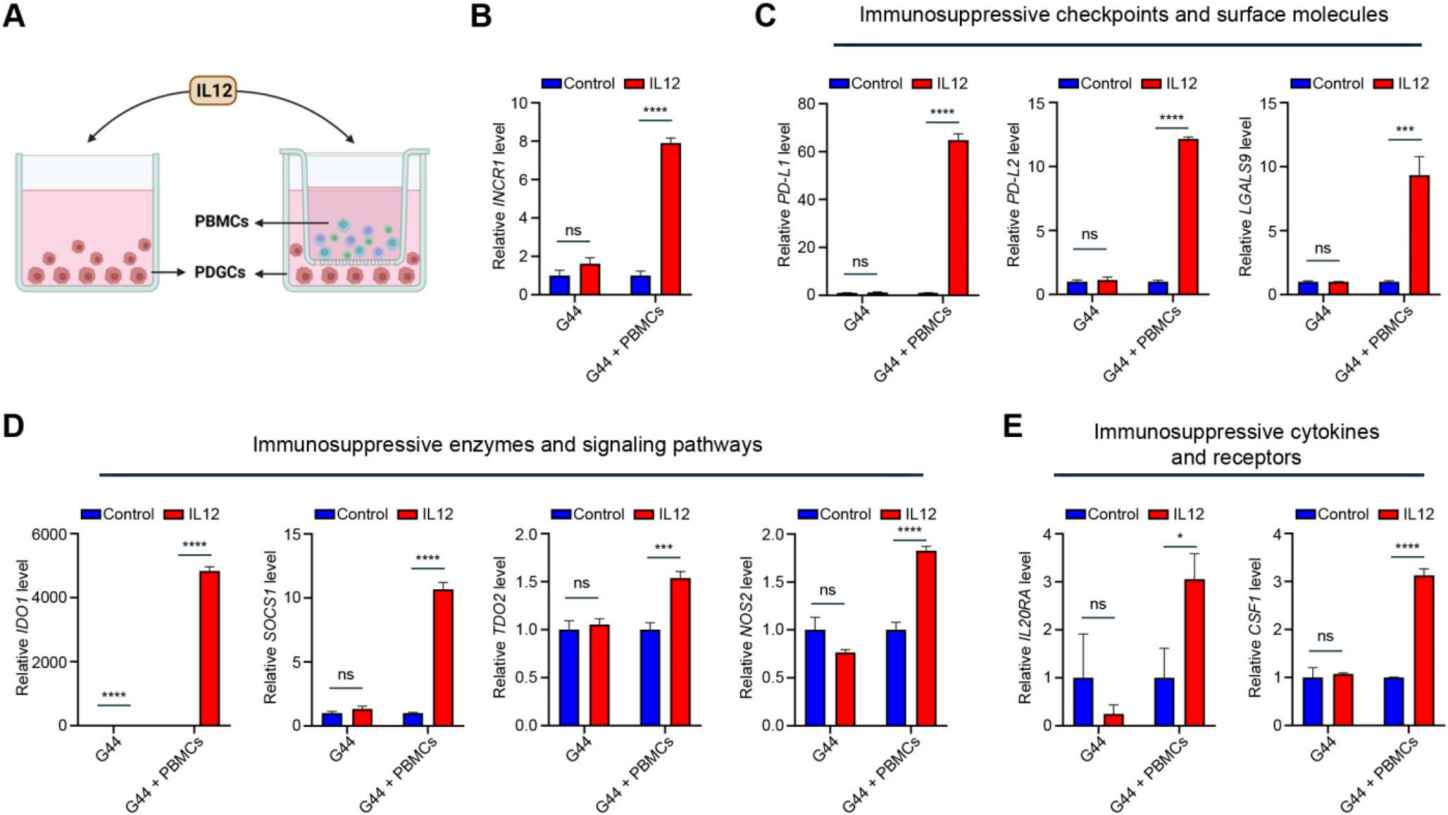
IL12 treatment induces the expression of multiple immunosuppressive genes in patient-derived GBM cells (PDGCs). **A** Schematic representation of the experimental design. PDGCs in monoculture (left) or in co-culture with PBMCs were treated with 50 ng/ml IL12 for 72 h. **B**-**E** qPCR analysis of *INCR1* (**B**), immunosuppressive checkpoints and surface molecules *PD-L1*, *PD-L2*, and *LGALS9* (**C**), immunosuppressive enzymes and signaling pathways *IDO1*, *SOCS1*, *TDO2*, and *NOS2* (**D**), immunosuppressive cytokines and receptors *IL20RA* and *CSF1* (**E**) in unstimulated or IL12 stimulated cells. Data shown as mean ± SD of three replicates. Data were analyzed by unpaired t-test: *p < 0.05, ***p < 0.001, ****p < 0.0001

IL12 is a potent immunostimulatory cytokine mainly produced by antigen-presenting cells (APCs). IL12 promotes activation and antitumor functions of natural killer (NK) cells, differentiation of CD4 + T cells to T helper 1 phenotype, and activation of cytotoxic T cells [11]. Activated NK and T cells secrete IFNγ, which is the major mediator of IL12-induced effects [11]. However, tumor cells respond to increased levels of IFNγ in the microenvironment by upregulating immunosuppressive genes [23]. Therefore, repeated administration of IL12 could induce the immunosuppressive properties of the tumors [24]. To identify immunosuppressive genes activated by IL12 in GBM, we cultured two different patient-derived GBM cells (PDGCs, G44 and BT139) in a monoculture or co-culture with peripheral blood mononuclear cells (PBMCs), and we treated them with IL12 (Fig. 2A). In both PDGCs, *INCR1* was upregulated in response to IL12 treatment when tumor cells were co-cultured with PBMCs (Fig. 2B and Fig. S2A). No significant effect of IL12 on *INCR1* expression was observed when G44 cells were cultured in the absence of PBMCs (Fig. 2B). A small but significant upregulation of *INCR1* was observed in BT139 monoculture treated with IL12 (Fig. S2A). In the co-culture condition, we also found the upregulation of several immunosuppressive genes in response to the IL12 treatment (Fig. 2C-E and Fig. S2B-D). Among those genes were immunosuppressive checkpoints and surface molecules (*PD-L1*, *PD-L2*, *LGALS9*, *FAS*, Fig. 2C and Fig. S2B), immunosuppressive enzymes and signaling pathways (*IDO1*, *SOCS1*, *TDO2*, *NOS2*, Fig. 2D and Fig. S2C), and immunosuppressive cytokines and receptors (*IL20RA*, *CSF1*, Fig. 2E and Fig. S2D). Except for *IDO1*, no significant effect of IL12 on the immunosuppressive gene expression was observed in G44 monoculture (Fig. 2C-E). A small increase in *PD-L2* and *FAS* expression was found in IL12-treated BT139 monoculture (Fig. S2B).

### Silencing *INCR1* leads to increased PBMC-mediated tumor cell killing upon IL12 treatment

**Fig. 3:**
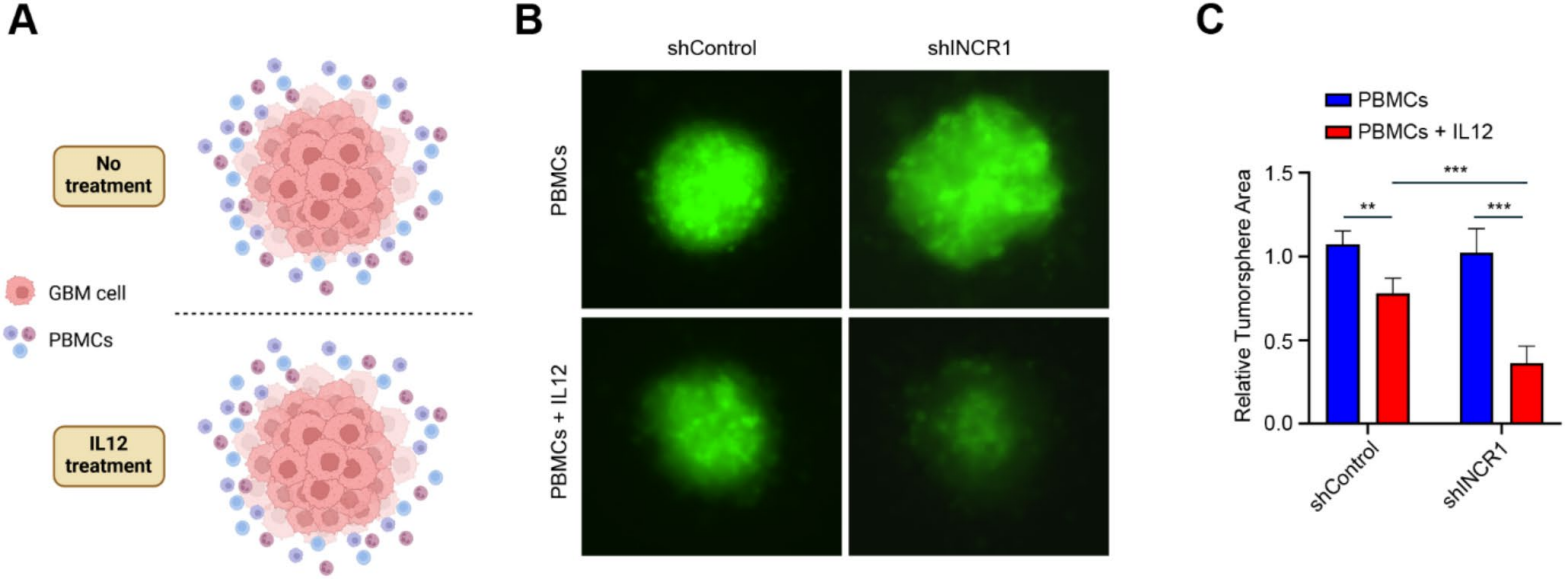
Silencing *INCR1* leads to increased PBMC-mediated tumor cell killing upon IL12 treatment. **A** Schematic representation of the experimental design. U251 stable transfected with shRNA control or an shRNA targeting *INCR1* were cultured as tumor spheres and co-cultured with PBMCs and treated with 50 ng/ml IL12. **B**-**C** Representative fluorescent microscopy pictures of GFP-positive control and *INCR1*-knockdown U251 tumor spheres co-cultured with PBMCs for 96 h with or without IL12 treatment (**B**) and relative tumor sphere area (**C**). Data shown as mean ± SD of three replicates. Data were analyzed by unpaired t-test: **p < 0.01, ***p < 0.001

PBMCs are composed of lymphocytes (B-cells, T cells, and NK cells; 70–90%), monocytes (10–20%), and dendritic cells (1–2%) [25]. Due to the overwhelming abundance of T cells (70–85%) within the PBMCs [25], these cells provide a useful model to study the effect of IL12 in vitro. Since we previously found that *INCR1* regulates the expression of several immunosuppressive genes [19], we hypothesized that silencing *INCR1* would increase PBMCs’ cytotoxic activity against tumor cells in response to IL12 stimulation. To test our hypothesis, we utilized our previously established stable *INCR1* knockdown U251 cell line. Control and stable *INCR1* knockdown cells were allowed to form 3D tumor spheroids on an ultralow attachment plate and then co-cultured with unstimulated or IL12-stimulated PBMCs (Fig. 3A). While IL12 treatment activated the PBMC cytotoxic effects against both control and *INCR1* knockdown tumor cells, we found a significant increase in the cytotoxicity of PBMCs on *INCR1* knockdown tumor cells compared to control (Fig. 3B-C). These results suggest that *INCR1* may represent a valid target to improve the efficacy of IL12 therapy.

### *INCR1* is a better target to improve IL12 immunotherapy compared to PD-1/PD-L1

**Fig. 4:**
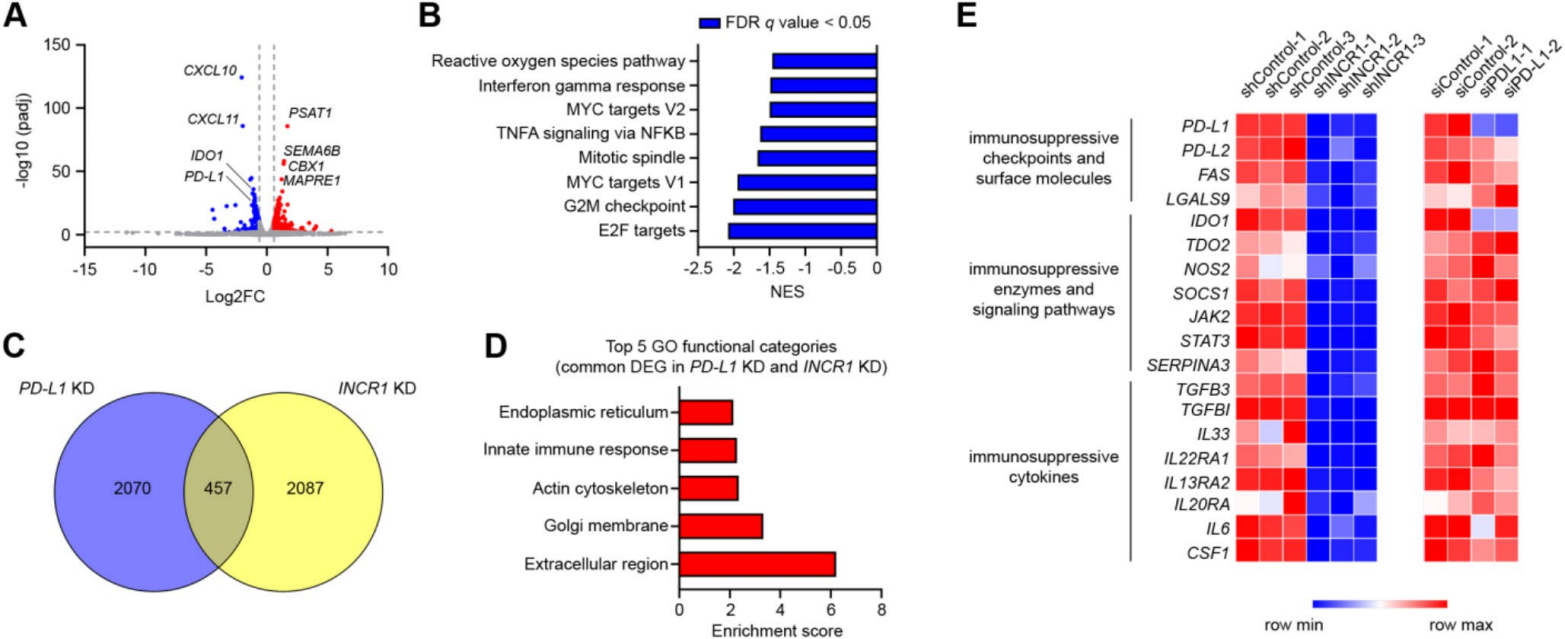
Knocking down *INCR1* reduced the expression of a larger number of immunosuppressive genes compared to *PD-L1* silencing. **A** Volcano plot of differentially expressed mRNAs (RNA-seq) between U251 cells transfected with an siRNA control and U251 cells transfected with two different siRNAs targeting *PD-L1*. Differentially expressed genes (DEGs) with an FDR < 0.01 and a fold-change ≥ 1.5 are depicted in red (up-regulated) and blue (down-regulated). **B** GSEA Hallmark analysis of enriched gene sets in *PD-L1* knockdown cells compared to control cells (FDR q-value < 0.05). A negative NES indicates enrichment in the control cells. **C** The Venn diagram shows the number of DEGs in *PD-L1* knockdown cells relative to *INCR1* knockdown cells. **D** Gene ontology analysis of genes commonly deregulated in *INCR1* knockdown cells compared to *PD-L1* knockdown cells. Analysis was performed using DAVID Bioinformatics Tool. **E** Heatmap of the expression levels of immunosuppressive genes in *INCR1* knockdown cells compared to control (*n* = 3 biological replicates) and *PD-L1* knockdown cells (*n* = 2 biological replicates)

To evaluate if *INCR1* is a better target than PD-L1 to improve IL12 therapy, we first assessed the impact of *PD-L1* silencing on global transcriptomic changes by RNA sequencing. Since PD-L1 expression is upregulated in response to IFNγ, U251 cells were transfected with an siRNA control or two different siRNAs targeting *PD-L1*, followed by stimulation with IFNγ. Knocking down *PD-L1* altered the expression of 752 genes (FDR < 0.01, fold change > 1.5, Fig. 4A and Table S3). Among the most significantly downregulated genes, we found the IFNγ-responding chemokines *CXCL10* and *CXCL11*, and the immunosuppressive enzyme *IDO1* (Fig. 4A). Gene set enrichment analysis (GSEA) of normalized counts of genes expressed in *PD-L1* knockdown compared to control showed 8 gene sets significantly downregulated in cells with silenced *PD-L1* and no significantly enriched gene sets (FDR < 0.05, Fig. 4B). The downregulated gene sets included “E2F targets”, “G2M checkpoint”, “Mitotic spindle”, “MYC targets V1 and V2” (Fig. 4B). These gene sets encompassed genes related to cell proliferation, confirming previous finding showing that silencing *PD-L1* in tumor cells reduced their growth [26]. The other downregulated gene sets were “TNFA signaling via NFkB”, “interferon-gamma response”, and “reactive oxygen species pathway” (Fig. 4B). By analyzing our previously published RNA sequencing data on IFNγ-stimulated U251 *INCR1* knockdown cells, we identified 457 commonly deregulated genes between *PD-L1* knockdown and *INCR1* knockdown cells (Fig. 4C). Gene ontology (GO) enrichment analysis of the common differentially expressed genes showed that the most enriched functional category was “extracellular space, which included genes that encode for secreted molecules (Fig. 4D). Other enriched GO terms were “Golgi membrane”, “actin cytoskeleton”, “innate immune response”, and “endoplasmic reticulum” (Fig. 4D). Notably, analysis of the expression of immunosuppressive genes revealed that while *PD-L1* silencing showed co-downregulation of only *IDO1*, silencing of *INCR1* resulted in the downregulation of multiple immunosuppressive genes, including immune checkpoints, immunosuppressive enzymes and cytokines (Fig. 4E).

**Fig. 5:**
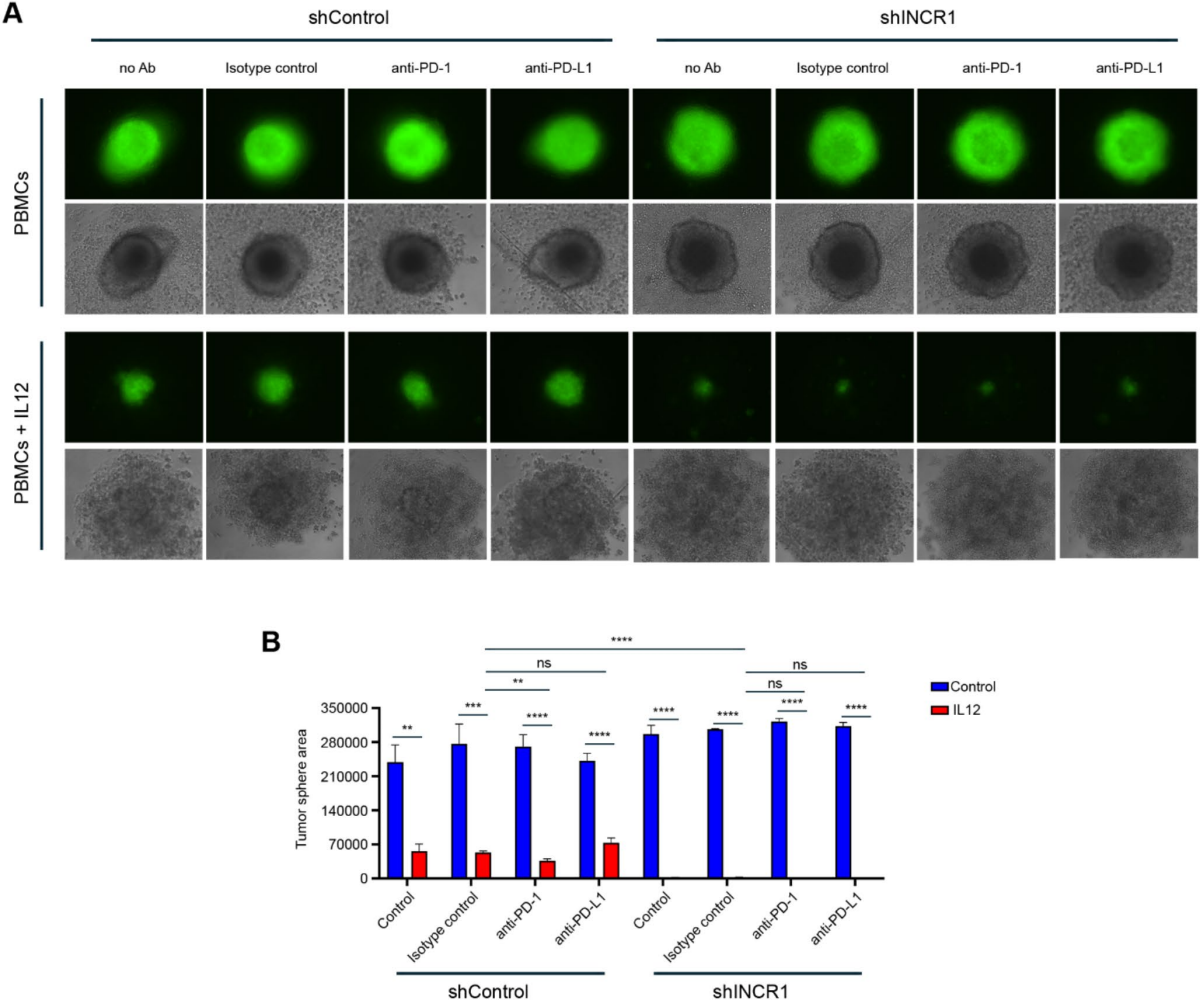
Silencing *INCR1* promotes an increased IL12-mediated PBMC cytotoxicity activity against tumor cells than antibodies against PD-1 or PD-L1. **A**-**B** Representative fluorescent microscopy pictures of GFP-positive control (left) and *INCR1*-knockdown (right) U251 tumor spheres co-cultured with PBMCs for 5 days and treated with 50 ng/ml IL12, 10 µg/ml anti-PD-1, 10 µg/ml anti-PD-L1 (**A**) and tumorsphere area analysis (**B**). Data shown as mean ± SD of three replicates. Data were analyzed by unpaired t-test: **p < 0.01, ***p < 0.001, ****p < 0.0001

To confirm that targeting *INCR1* could improve IL12 therapy compared to the inhibition of the PD-1/PD-L1 signaling, we used our 3D culture system (Fig. 3A) to co-culture control and *INCR1* knockdown cells with IL12-treated PBMCs in the presence of antibodies targeting PD-1 or PD-L1 immune checkpoint (Fig. 5A). We found that IL12 treatment promoted the cytotoxic activity of the PBMCs in all the conditions tested. However, while the anti-PD-1 antibody had a small effect on improving PBMC-mediated cytotoxicity, silencing *INCR1* promoted a greater activity of PBMCs against the tumorspheres (Fig. 5A-B). No difference in PBMC cytotoxicity was observed with the anti-PD-L1 antibody or the combination of *INCR1* silencing with anti-PD-1/PD-L1 antibodies (Fig. 5B). Taken together, our results show that silencing *INCR1* reduces the expression of a more significant number of immunosuppressive genes compared to *PD-L1* and suggest *INCR1* as a better target to improve IL12 therapy in GBM.

### Targeting *INCR1* using ASOs reduces the expression of multiple immunosuppressive molecules

Our results suggest that *INCR1* is a promising target for immunotherapy. *INCR1* can be targeted in GBM patients with chemically modified antisense oligonucleotides (ASOs), which induce an RNAse H-dependent degradation of the target RNA [27]. ASOs are currently used in the clinic for the treatment of a variety of neurological disorders, and the route of ASO delivery to the brain is well established [28–31]. Therefore, we designed and tested three LNA gapmer ASOs against *INCR1*. Since IFNγ is the major mediator of IL12-induced effects [11], PDGCs were transfected with ASOs and then treated with IFNγ for 48 h. Of the three ASOs analyzed, we found that ASO#3 was effective in reducing *INCR1* expression compared to a negative control ASO in both control and IFNγ-treated PDGCs (Fig. S3A). Notably, targeting *INCR1* with ASO#3 reduced the expression of immunosuppressive genes, including immunosuppressive checkpoints and surface molecules (*PD-L1*, *PD-L2*, Fig. S3B), immunosuppressive enzymes and signaling pathways (*IDO1* and *TDO2*, Fig. S3C), and the immunosuppressive cytokine *CSF1* (Fig. S3D). Together, these results suggest ASOs as promising therapeutic tools to target *INCR1* in patients.

## Discussion

IL12 is a potent immunostimulatory cytokine with a critical role in regulating the antitumor immune responses [11]. IL12 is mainly produced by antigen-presenting cells (APCs) and promotes the activation and antitumor functions of natural killer (NK) and T cells [32]. Mice lacking IL12 were shown to develop spontaneous tumors at a higher frequency than wild-type mice [33, 34]. Moreover, in humans, polymorphisms in the *IL12A* gene were associated with an increased susceptibility to developing GBM [35]. While IL12 showed great potential in several different preclinical models, i.v. injection of IL12 in cancer patients not only demonstrated poor efficacy but was also associated with cytokine release syndrome (CRS) [11, 36–38]. These results led to the development of alternative strategies for the local delivery of diverse IL12 gene therapies that could reduce the severe adverse effects [11]. We have recently conducted a clinical trial of peritumoral administration of a regulatable adenoviral vector encoding human IL12 (Ad-RTS-hIL-12) in patients with rGBM [13]. We showed that the therapy was well tolerated, and we found an increased number of immune infiltrates and IFNγ expression that lasted several months post-IL12 therapy [13]. However, the patient median overall survival of 12.7 months suggested that the persistent activation of the IFNγ signaling in GBM cells could have promoted the expression of immunosuppressive molecules that generated a negative feedback signal and reduced immune cell function. In this study, we showed that tumor tissues from GBM patients treated with IL12 gene therapy presented an upregulation of the lncRNA *INCR1* compared to the tumor tissues of the same patients before the IL12 treatment. Increased expression of *INCR1* was associated with concomitant upregulation of the immune checkpoint mRNA *PD-L1*. We also showed that IL12 treatment of patient-derived GBM cells co-cultured with PBMCs stimulated *INCR1* expression and the upregulation of *PD-L1* and several other immunosuppressive molecules, including immune checkpoints, immunosuppressive enzymes, and immunosuppressive cytokines.

*INCR1* is a novel discovered lncRNA transcribed from the *PD-L1* locus [19]. *INCR1* and PD-L1 are two molecules with strong immunosuppressive functions. PD-L1 acts as an immune inhibitory receptor ligand that is expressed by hematopoietic and non-hematopoietic cells [39]. The binding of PD-L1 to the immune checkpoint receptor PD-1 on the surface of effector T cells induces PD-1 phosphorylation and inhibition of T cell growth and cytokine secretion [40]. Systemic monoclonal antibodies against PD-1 and PD-L1 are widely used and have produced durable tumor regression and overall survival benefits in several solid and hematological cancers [41, 42]. Based on this, a follow-up clinical trial was designed to determine the tolerability and efficacy of neo-adjuvant anti-PD-1 combined with IL12 gene therapy in GBM. Unexpectedly, the combination therapy showed no difference in tolerability or efficacy compared to the IL12 gene monotherapy trial [14]. These results suggested that GBM immune evasion is complex and may depend on multiple signaling pathways, not just the PD-1/ PD-L1 pathway. Since we have recently discovered that silencing *INCR1* reduced the expression of different immunosuppressive genes [19], we hypothesized that targeting *INCR1* could represent an alternative strategy to improve IL12 immunotherapy. Using a 3D co-culture system, we demonstrated that GBM cells with silenced *INCR1* were more susceptible to PBMC-mediated killing in the presence of IL12 compared to control cells. One possible reason for the increased PBMC cytotoxic effects is reduced T-cell exhaustion when *INCR1* is silenced in tumor cells. In fact, we have previously shown in an immunodeficient GBM mouse model treated with CAR T cells that, compared to control tumors, tumors with silenced *INCR1* presented a higher number of CD4 + T cells infiltrated and reduced expression of PD-1 in CD8 + T cells [19]. Notably, global transcriptomic analysis of *INCR1* knockdown and *PD-L1* knockdown GBM cells revealed that silencing *INCR1* reduced the expression of a significantly larger number of immunosuppressive molecules compared to *PD-L1* silencing, which showed a reduction of only itself and the immunosuppressive enzyme *IDO1*. More than 90% of GBM expresses IDO1 [43], and its expression was associated with poor prognosis in patients with glioma [44]. IDO1 is an enzyme that metabolizes the essential amino acid tryptophan into kynurenine. Kynurenine secreted by tumor cells inhibits T cell activation and proliferation and induces Forkhead box P3 (FoxP3) expression, which is critical for the differentiation and function of Treg cells [45]. While we showed that silencing *PD-L1* using siRNAs reduced the expression of IDO1, upregulation of IDO1 has been proposed as a mechanism of resistance to anti-PD-1 treatment [46]. Inhibition of IDO1 synergized with anti-PD-1 and radiation in a mouse model of GBM [47]. By targeting *INCR1*, we could concomitantly block *IDO1*, *PD-L1*, and other immunosuppressive molecules. Most importantly, *INCR1* silencing improved IL12-mediated PBMC cytotoxicity against tumor cells compared to monoclonal antibodies targeting PD-1 or PD-L1.

One limitation of our study is the lack of an immunocompetent mouse model to validate these data in vivo further. This is primarily due to the minimal sequence conservation of lncRNAs across species [48]. The use of mouse GBM cells will require an extensive lncRNA analysis to identify the sequence and validate the function of mouse *INCR1*. Even if our 3D co-culture system provides a physiological cell behavior that mimics tumor biology, an alternative future strategy would be the study of *INCR1* and combination therapy in human GBM organoids. Another possibility would be the development of a humanized mouse model. The generation of a mouse model will also be important in performing safety studies on combining *INCR1* inhibition with IL12. Our data suggest that *INCR1* can be targeted in patients using ASOs. Transfection of ASOs against *INCR1* in PDGCs reduced the expression of *INCR1* and other immunosuppressive molecules. Our clinical trial aimed at determining the tolerability and efficacy of neo-adjuvant nivolumab (anti-PD-1) combined with IL12 gene therapy in GBM showed that the combination was well tolerated, with a toxicology profile comparable to IL12 gene monotherapy [14]. While we do not expect ASOs targeting *INCR1* to exacerbate IL12-related toxicity, future pre-clinical studies will be needed to evaluate the safety of this combination therapy.

In conclusion, *INCR1* expression is induced in GBM patients treated with regulatable IL12 gene therapy. Targeting *INCR1* reduces more immunosuppressive molecules than *PD-L1* and increases tumor cell susceptibility to IL12-mediated immune cell cytotoxicity. Moreover, *INCR1* silencing enhanced the antitumor activity of IL12-stimulated immune cells compared to immune checkpoint inhibitors. Therefore, targeting *INCR1* represents a promising approach to improve the efficacy of IL12 immunotherapy in GBM and potentially other tumors.

## Electronic supplementary material

Below is the link to the electronic supplementary material.

Supplementary Material 1

Supplementary Material 2

## Data Availability

The datasets generated during and/or analyzed during the current study are available in the Gene Expression Omnibus (GEO), GSE137489, GSE285565.

## Code availability

No custom code has been used in this study.
