# Supplementary material for: Improving IL12 immunotherapy in glioblastoma by targeting the long noncoding RNA INCR1
Source: J Neurooncol. 2025 Mar 4;173(1):205–16. doi: 10.1007/s11060-025-04978-2 (PMC12041012; doi:10.1007/s11060-025-04978-2)
Supplement: Supplementary file 1 — Supplementary Material 1 [file 11060_2025_4978_MOESM1_ESM.pdf]

Supplementary Data and Methods

Improving IL12 immunotherapy in glioblastoma by targeting the long noncoding RNA *INCR1*.

Shikha Saini, Josephina A.M.A. Gadet, Gordon J. Freeman, E. Antonio Chiocca, Marco Mineo

Table S1. Real-Time qPCR primers

**18S_Forward**	AACTTTCGATGGTAGTCGCCG
**18S_Reverse**	CCTTGGATGTGGTAGCCGTTT
**INCR1_Forward**	GTGGGATATGACAGGGACGC
**INCR1_Reverse**	GGCAGGAGCAGCTAATCCAA
**PD-L1_Forward**	GAGTGGTAAGACCACCACC
**PD-L1_Reverse**	GGTTTTCCTCAGGATCTAAT
**PD-L2_Forward**	AGTGCTATCTGAACCTGTGGTC
**PD-L2_Reverse**	AGTGCTGGGTCATCCAAAGG
**LGALS9_Forward**	CCCTACCTGAGTCCAGCTGTC
**LGALS9_Reverse**	ACGGTCCCATTGACAGTGAT
**IDO1_Forward**	TCTGGCCAGCTTCGAGAAAG
**IDO1_Reverse**	AGAACTAGACGTGCAAGGCG
**SOCS1_Forward**	CACTTCCGCACATTCCGTTC
**SOCS1_Reverse**	CAGTAGAATCCGCAGGCGTC
**TDO2_Forward**	CGGTGGTTCCTCAGGCTATC
**TDO2_Reverse**	CTTCGGTATCCAGTGTCGGG
**NOS2_Forward**	ATCTGCAGACACGTGCGTTA
**NOS2_Reverse**	CGGATGAGCTGAGCATTCCA
**CSF1_Forward**	TGAGACACCTCTCCAGTTGCTG
**CSF1_Reverse**	GCAATCAGGCTTGGTCACCACA
**FAS_Forward**	GGACCCAGAATACCAAGTGCAG
**FAS_Reverse**	GTTGCTGGTGAGTGTGCATTCC
**IL20RA_Forward**	CAAACTCCCCGAAAGGGTCA
**IL20RA_Reverse**	AGGCAAACCACCAGAGACAC

Table S2. Patient characteristics.

IDH, isocitrate dehydrogenase; MGMT, methylguanine methyltransferase.

Patient ID	***Age***	***Gender***	***Diagnosis***	***Disease setting***	***Prior bevacizumab***	***MGMT status***
PT10	49	F	GBM, IDH-WT	Recurrent	Yes	Unmethylated
PT17	34	M	GBM, IDH-WT	Recurrent	Yes	Methylated
PT37	46	M	GBM, IDH-WT	Recurrent	Yes	Methylated
PT38	46	F	GBM, IDH-WT	Recurrent	No	Unmethylated
PT39	67	M	GBM, IDH-WT	Recurrent	Yes	Unmethylated

Fig. S1

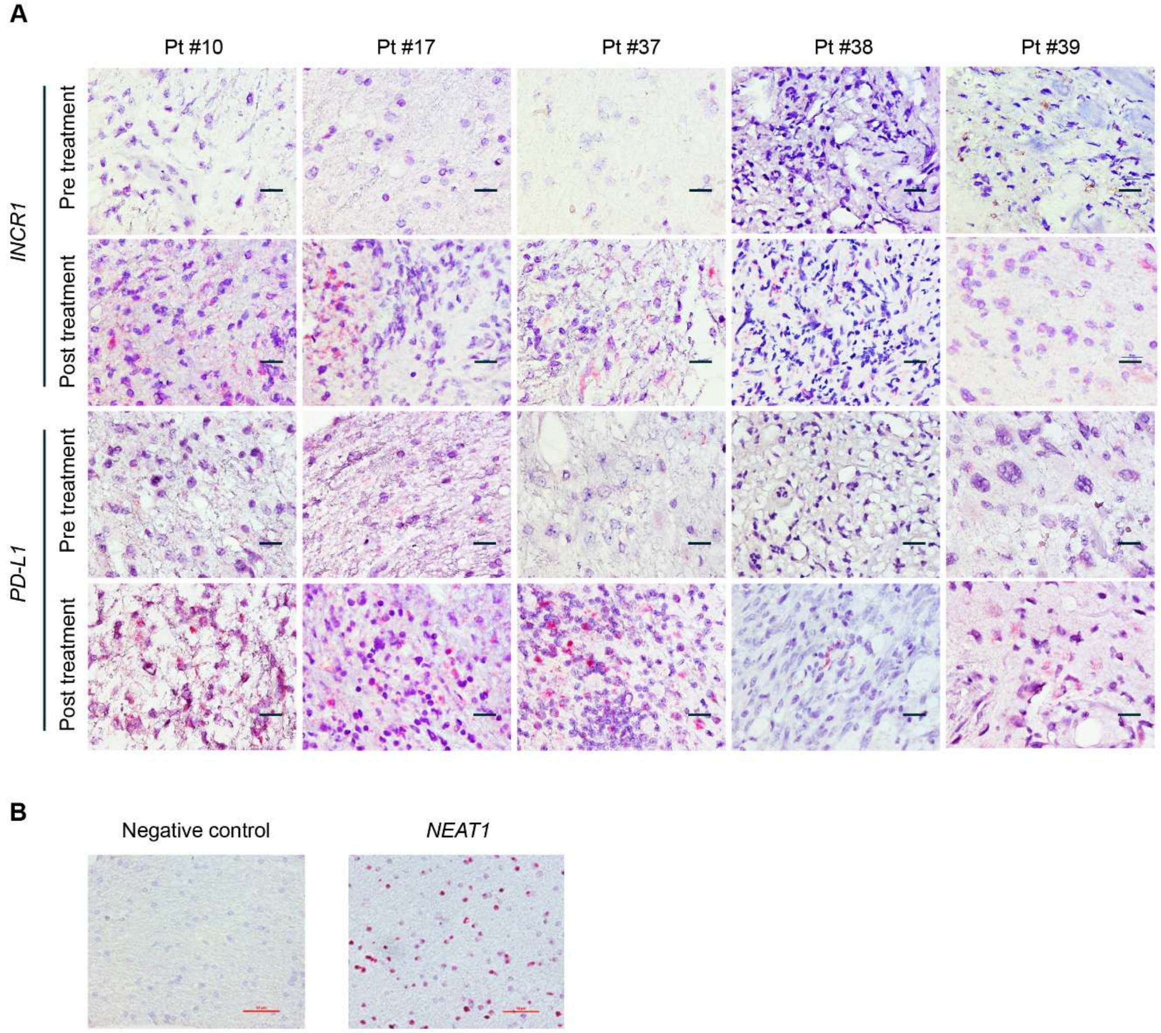

A RNAscope *in situ* hybridization analysis of *INCR1* (top) and *PD-L1* (bottom) expression in GBM tumors pre- and post-IL12 gene therapy. Red dots are indicative of *INCR1* and *PD-L1* signals. Scale bar: 20 µm. **B** RNAscope *in situ* hybridization analysis using a negative control probe (left) and a probe targeting the lncRNA *NEAT1* (right). Red dots are indicative of *NEAT1* signals. Scale bar: 50 µm.

Fig. S2

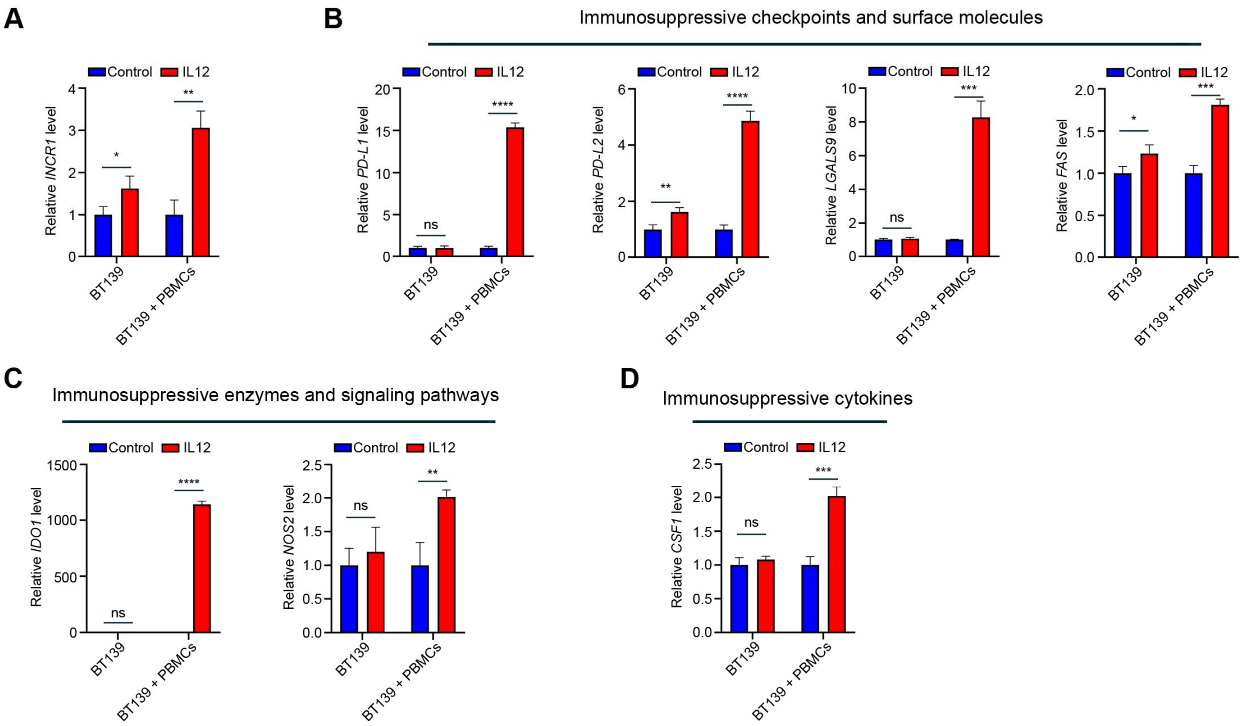

IL12 treatment induces the expression of multiple immunosuppressive genes in patient-derived GBM cells (PDGCs). **A-D** qPCR analysis of *INCR1* (**A**), immunosuppressive checkpoints and surface molecules *PD-L1*, *PD-L2*, *LGALS9*, and *FAS* (**B**), immunosuppressive enzymes and signaling pathways *IDO1*, and *NOS2* (**C**), immunosuppressive cytokine *CSF1* (**D**) in unstimulated or IL12 stimulated BT139 cells. Data shown as mean ± SD of three replicates. Data were analyzed by unpaired t-test: *p < 0.05, **p < 0.01, ***p < 0.001, ****p < 0.0001.

Fig. S3

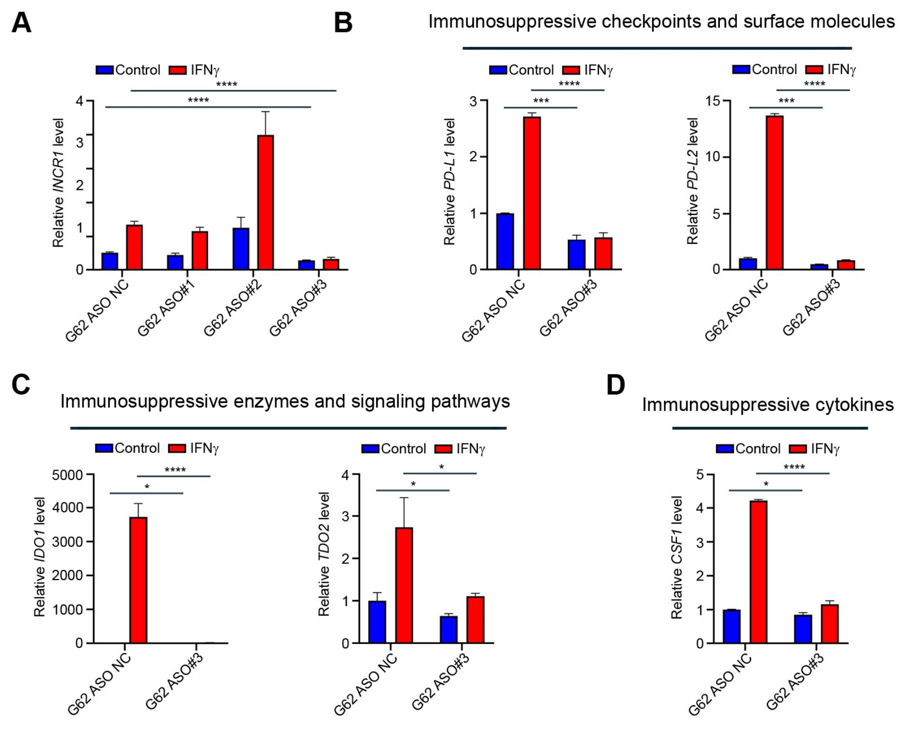

Antisense oligonucleotides (ASOs) targeting *INCR1* reduced the expression of multiple immunosuppressive genes in patient-derived GBM cells. **A** qPCR analysis of *INCR1* expression in unstimulated or IFNγ stimulated G62 cells transfected with an ASO negative control (NC) or three different ASOs targeting *INCR1*. **B-D** qPCR analysis of immunosuppressive checkpoints and surface molecules *PD-L1*, and *PD-L2* (**B**), immunosuppressive enzymes and signaling pathways *IDO1*, and *TDO2* (**C**), immunosuppressive cytokine *CSF1* (**D**) in unstimulated or IFNγ stimulated G62 cells transfected with an ASO NC or *INCR1* ASO#3. Data shown as mean ± SD of three replicates. Data were analyzed by unpaired t-test: *$p < 0.05$, ***$p < 0.001$, ****$p < 0.0001$.
